# Supplementary material for: Effect of Different Crowding Agents on the Architectural Properties of the Bacterial Nucleoid-Associated Protein HU
Source: Int J Mol Sci. 2020 Dec 15;21(24):9553. doi: 10.3390/ijms21249553 (PMC7765392; doi:10.3390/ijms21249553)
Supplement: Supplementary file 1 [file ijms-21-09553-s001.pdf]

Supplementary information

SI figure 1 to 5 include multiple independent experiments which are labelled with different colours and patterns.

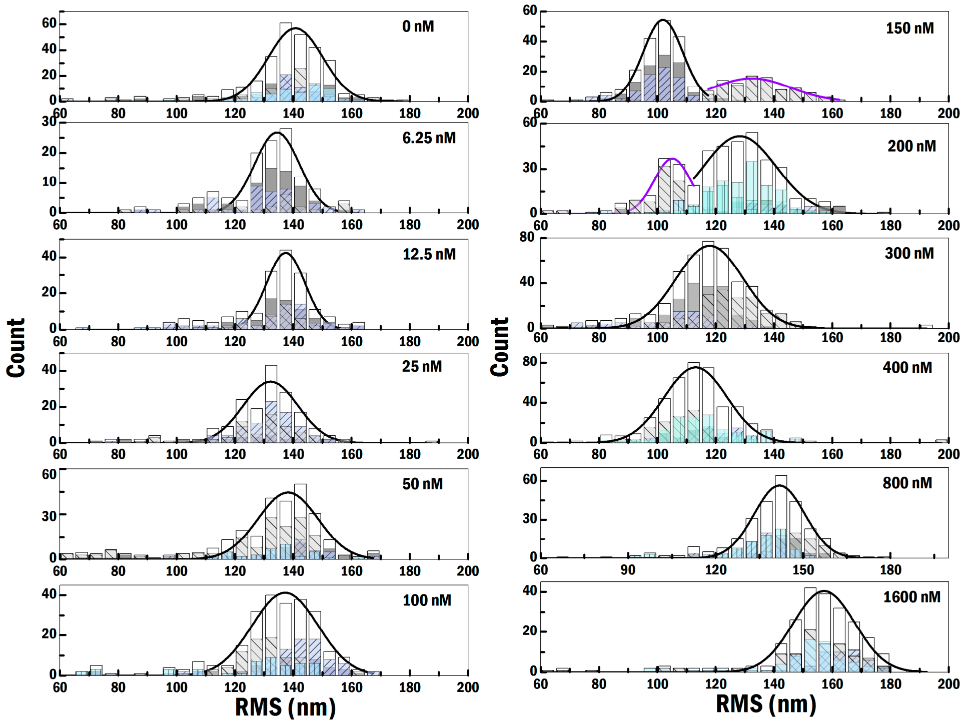

SI Figure 1A. Distribution of RMS values obtained at different HU concentration in the absence of $MgCl_2$.

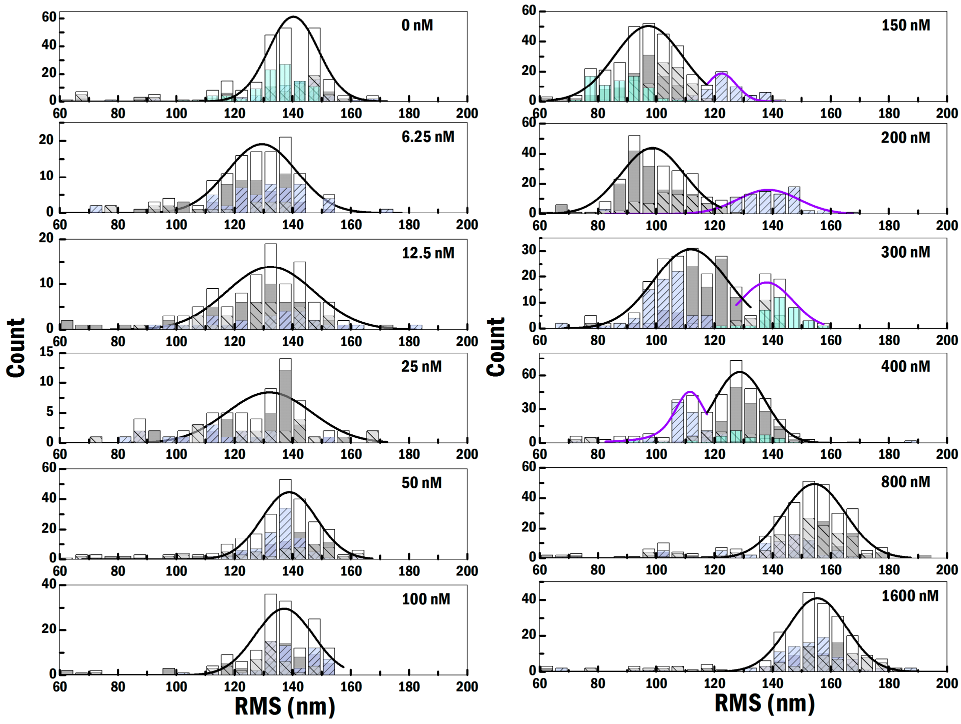

SI Figure 1B. Distribution of RMS values obtained at different HU concentration in the presence of $MgCl_2$.

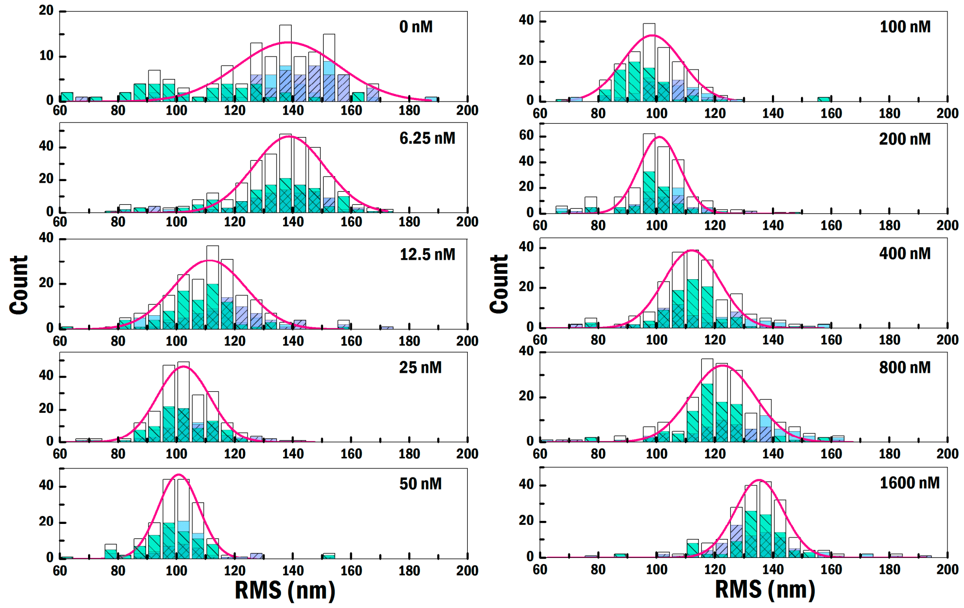

SI Figure 2A. Distribution of RMS values obtained at different HU concentration in the presence of 0.5 % (w/v) BGB in the absence of $MgCl_2$.

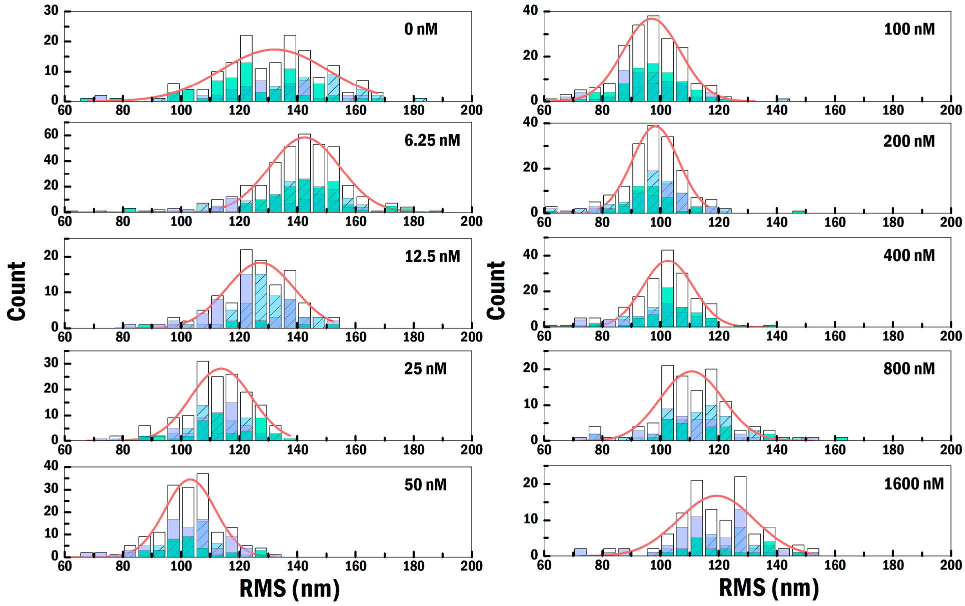

SI Figure 2B. Distribution of RMS values obtained at different HU concentration in the presence of 1 % (w/v) BGB in the absence of $MgCl_2$.

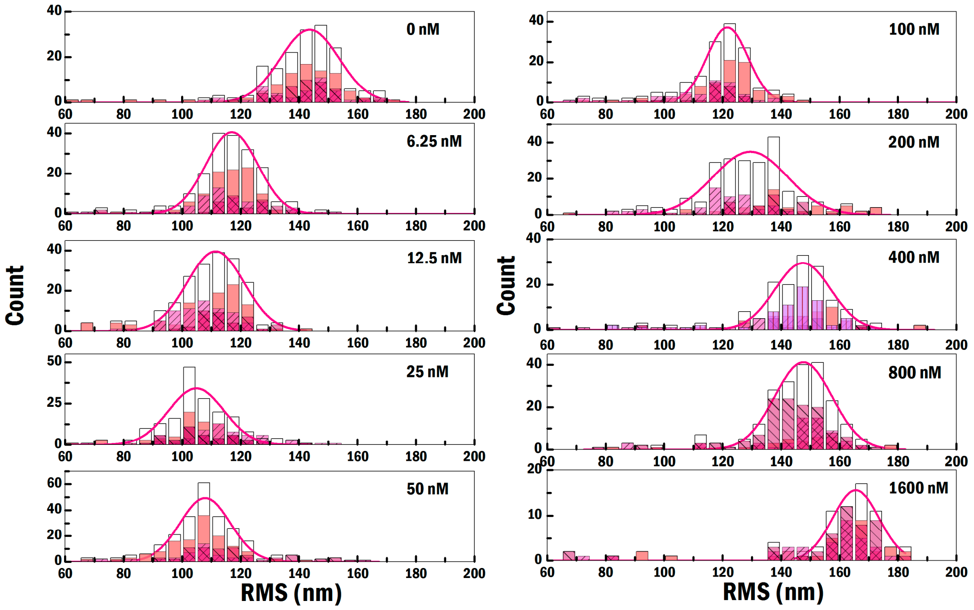

SI Figure 3A. Distribution of RMS values obtained at different HU concentration in the presence of 1.25 % (w/v) BSA in the absence of $MgCl_2$.

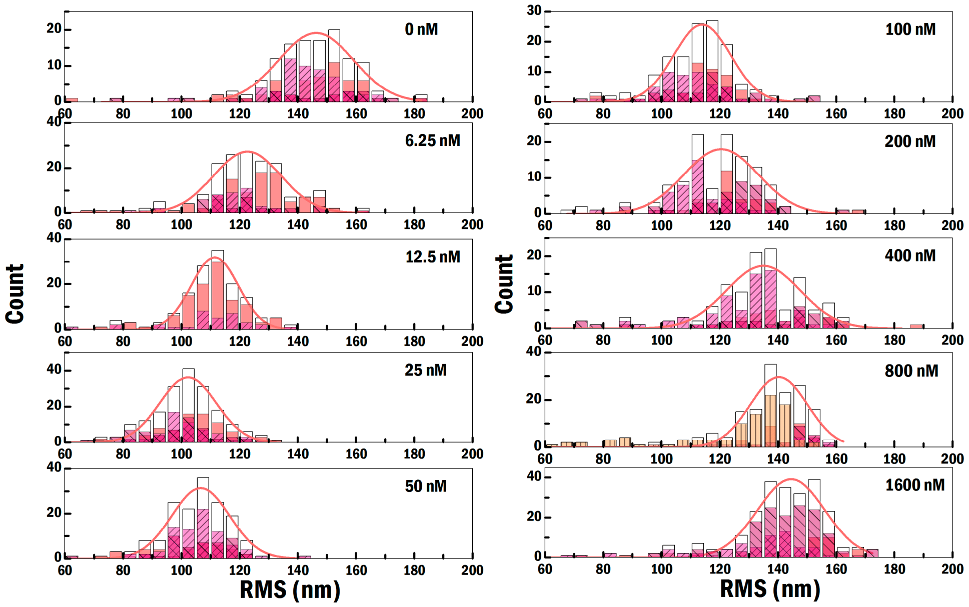

SI Figure 3B. Distribution of RMS values obtained at different HU concentration in the presence of 5 % (w/v) BSA in the absence of $MgCl_2$.

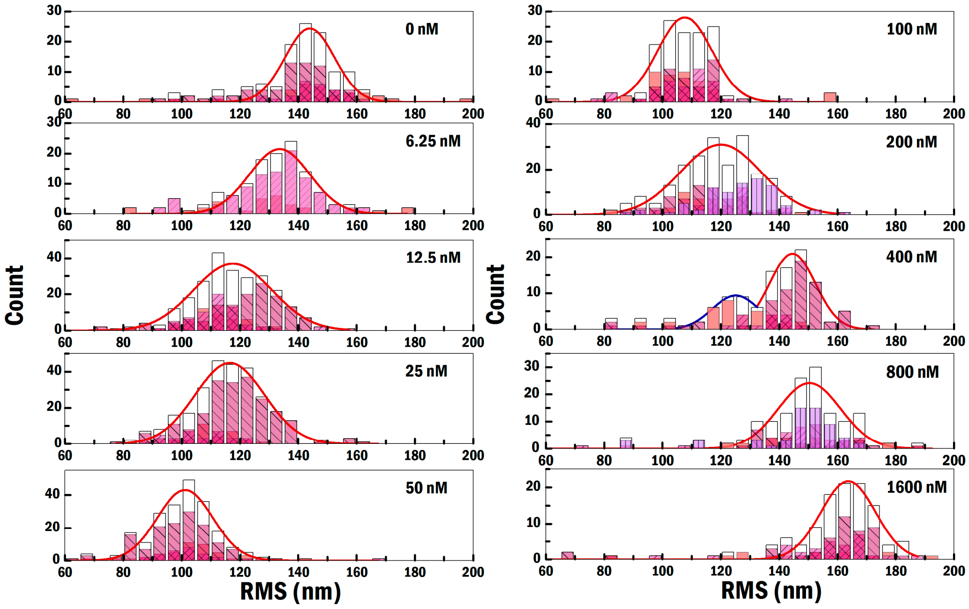

SI Figure 3C. Distribution of RMS values obtained at different HU concentration in the presence of 10 % (w/v) BSA in the absence of $MgCl_2$.

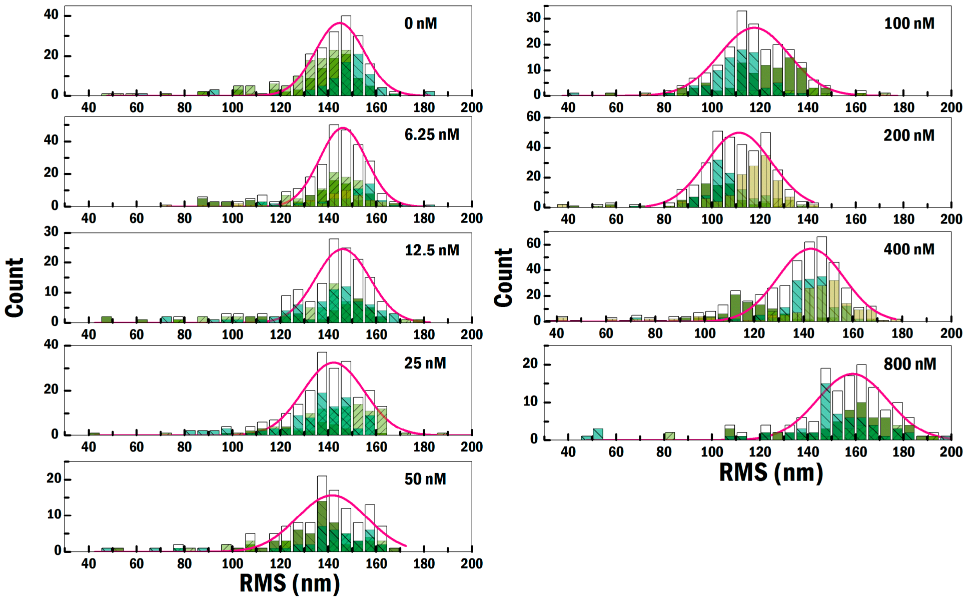

SI Figure 4A. Distribution of RMS values obtained at different HU concentration in the presence of 3 % (w/v) PEG8000 in the absence of $MgCl_2$.

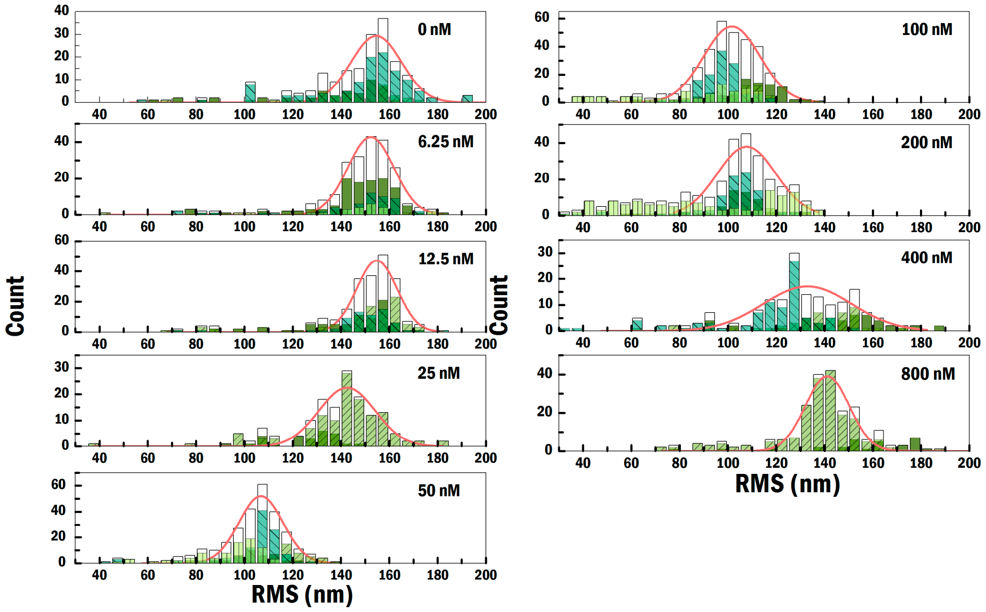

SI Figure 4B. Distribution of RMS values obtained at different HU concentration in the presence of 9 % (w/v) PEG8000 in the absence of $MgCl_2$.

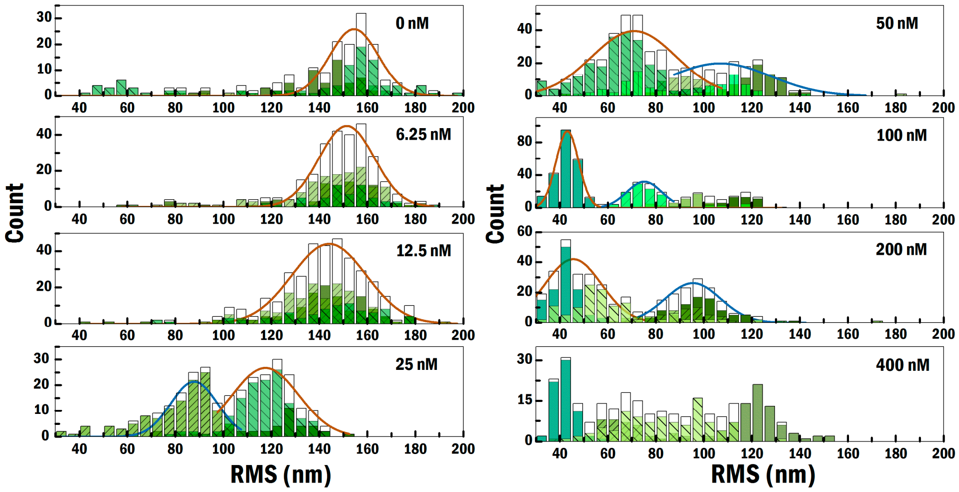

SI Figure 4C. Distribution of RMS values obtained at different HU concentration in the presence of 15 % (w/v) PEG8000 in the absence of $MgCl_2$.

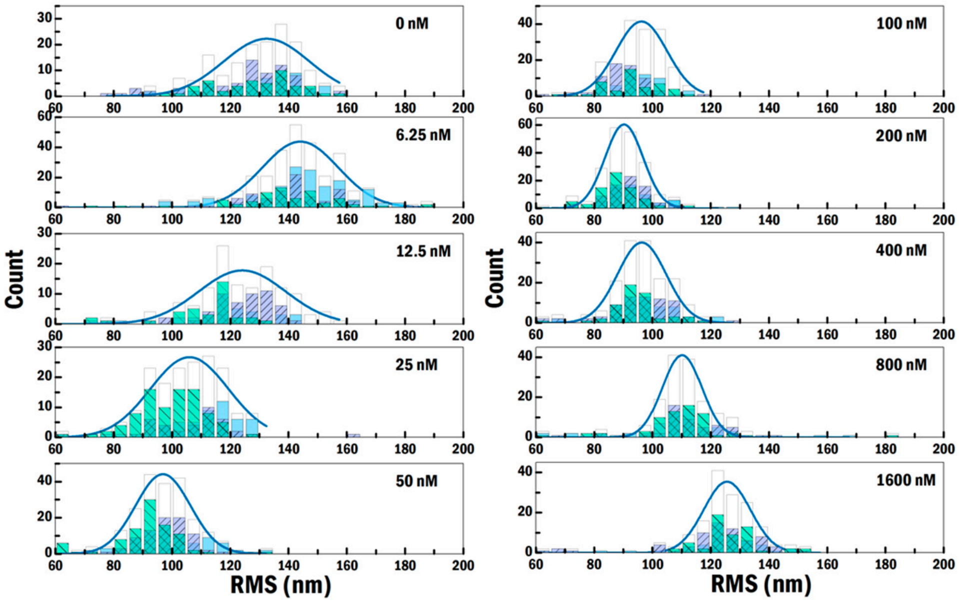

SI Figure 5A. Distribution of RMS values obtained at different HU concentration in the presence of 0.5 % (w/v) BGB in the presence of $MgCl_2$.

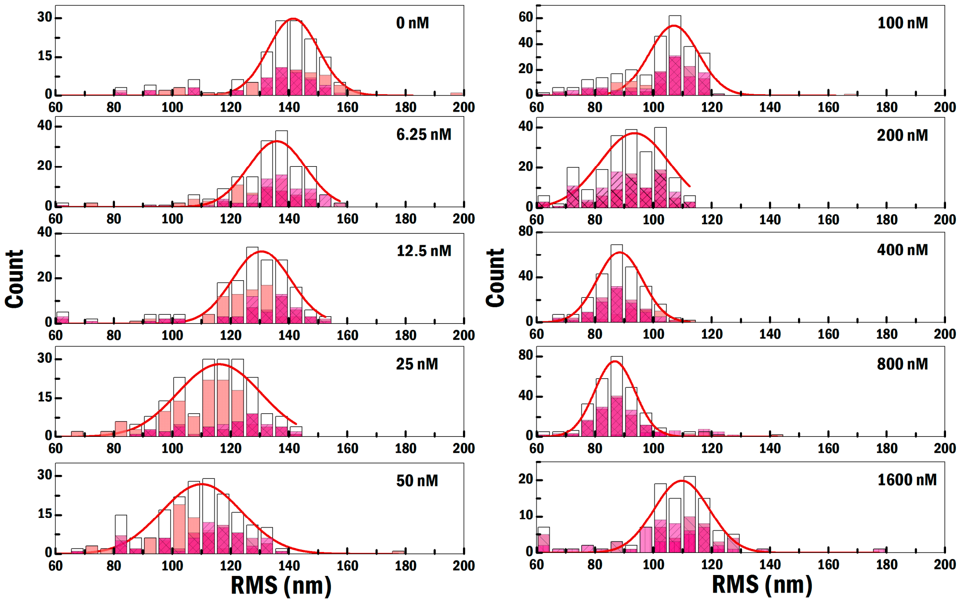

SI Figure 5B. Distribution of RMS values obtained at different HU concentration in the presence of 10 % (w/v) BSA in the presence of $MgCl_2$.

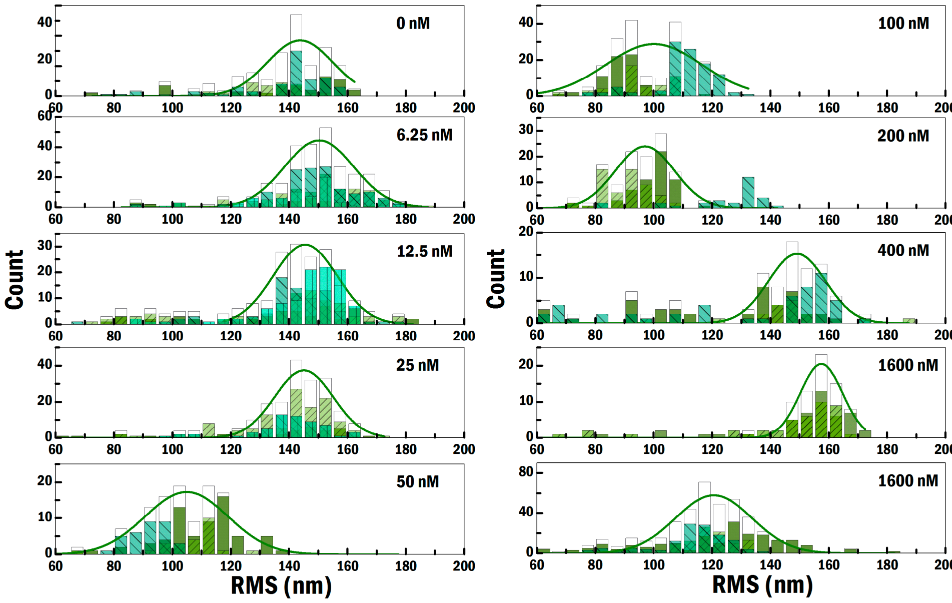

SI Figure 5C. Distribution of RMS values obtained at different HU concentration in the presence of 3 % (w/v) PEG8000 in the presence of $MgCl_2$.

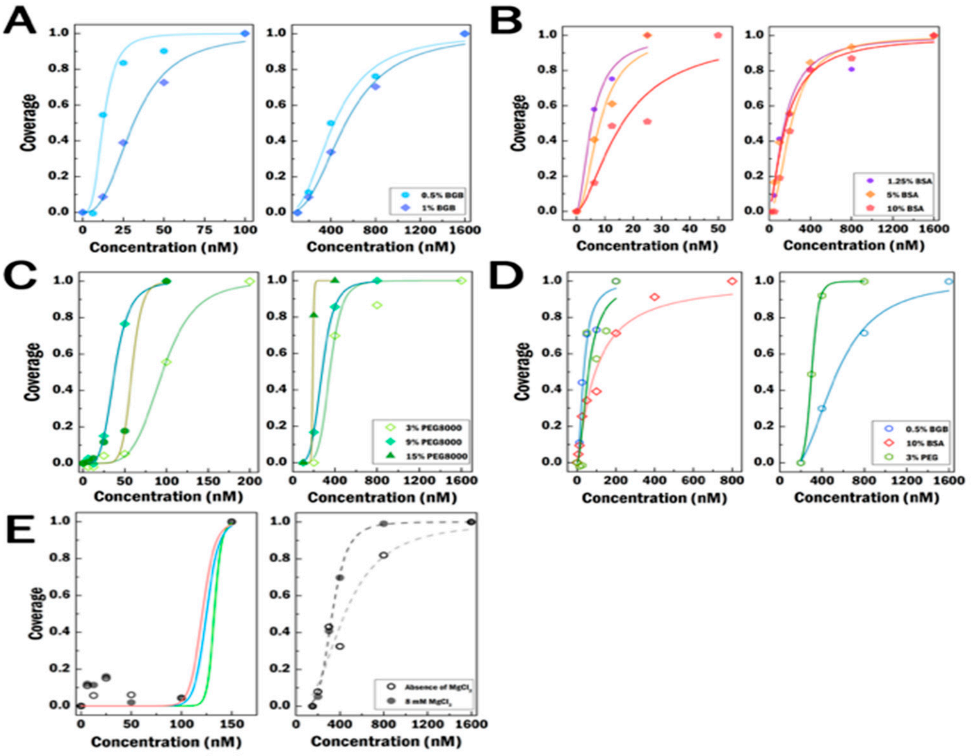

SI Figure 6. The fractional coverage as a function of HU concentration fitted by Hill function. A, B, C are the fittings of two HU binding modes in the absence of $MgCl_2$ and with BSA, BGB, and PEG8000, respectively. D are the fittings of two HU binding modes in the presence of $MgCl_2$ and with BSA, BGB, and PEG8000, respectively. E is in crowder-free condition in the presence (empty circle) or presence (solid circle) of $MgCl_2$. In the left plot of E shows theoretical curves are shown with cooperativity value of 19 $\pm$ 2 (red), 20 $\pm$ 2 (blue), and 38 $\pm$7 (green). For each plot: left) DNA binding behavior in the compaction regime; right) DNA binding behavior in the extension regime.

SI Table 1. The RMS values of HU-DNA in presence and absence of $MgCl_2$.

HU Conc. (nM)	Without $MgCl_2$*	N	With 8 mM $MgCl_2$**	N
**0**	140.8 ± 0.4	300	140.3 ± 0.6	302
**6.25**	134.5 ± 0.7	128	129 ± 1	72
**12.5**	137.5 ± 0.6	180	132 ± 1	116
**25**	132.3 ± 0.7	180	129 ± 1	126
**50**	137.2 ± 0.6	224	138.9 ± 0.7	245
**100**	138.3 ± 0.4	306	137.1 ± 1	158
**150**	102.4 ± 0.7	198	97.3 ± 0.8	222
	132 ± 1	198	122 ± 1	42
**200**	128.3 ± 0.8	261	99 ± 1	244
	105.2± 0.6	172	139 ± 1	88
**300**	118.0 ± 0.5	390	111.9 ± 0.6	190
			138 ± 3	58
**400**	113.4 ± 0.7	393	125 ± 1	265
			111.6 ± 0.8	34
**800**	141.9 ± 0.4	195	154.4 ± 0.7	305
**1600**	157.2 ± 0.4	161	155.3 ± 0.4	235

*Without $MgCl_2$: 10 mM Tris (pH 7.5), 50 mM NaCl, 1 mM EDTA
**Without 8 mM $MgCl_2$: 10 mM Tris (pH 7.5), 50 mM NaCl, 8 mM $MgCl_2$

SI Table 2. The RMS values of HU-DNA in presence of BGB in buffer containing and absence of Mg^{2+}.

HU Conc. (nM)	0.5 % BGB without $MgCl_2$	N	1 % BGB without $MgCl_2$	N	0.5 % BGB without $MgCl_2$	N
**0**	138 ± 2	134	132 ± 2	162	133 ± 2	171
**6.25**	138.8 ± 0.7	313	143 ± 0.6	385	144 ± 1	312
**12.5**	111.4 ± 0.9	201	127 ± 1	111	124 ± 2	118
**25**	102.5 ± 0.6	225	113.8 ± 0.9	153	106 ± 1	176
**50**	100.8 ± 0.4	183	103.2 ± 0.9	164	96.9 ± 0.6	227
**100**	98.5 ± 0.7	173	97 ± 0.4	191	96.2 ± 0.8	170
**200**	101 ± 0.6	246	98.3 ± 0.7	172	90.3 ± 0.4	22
**400**	112.1 ± 0.6	211	102.6 ± 0.05	175	96.4 ± 0.6	184
**800**	123 ± 1	211	111 ± 1	114	110.2 ± 0.5	171
**1600**	135.3 ± 0.4	205	119 ± 2	113	125.6 ± 0.6	162

SI Table 3. The RMS values of HU-DNA in presence of BSA in buffer containing and absence of Mg^{2+}.

HU Conc. (nM)	1.25 % BSA without $MgCl_2$	N	5 % BSA without $MgCl_2$	N	10 % BSA without $MgCl_2$	N	10 % BSA without $MgCl_2$	N
**0**	143.7 ± 0.5	176	146.3 ± 0.7	128	144 ± 0.6	127	141.5 ± 0.5	155
**6.25**	117.0 ± 0.3	198	125 ± 1	173	133.6 ± 0.8	244	135.9 ± 0.8	179
**12.5**	111.5 ± 0.6	207	101.8 ± 0.5	441	117.6 ± 0.7	249	130.6 ± 0.7	188
**25**	105 ± 1	183	102.3 ± 0.5	190	116.5 ± 0.6	277	116 ± 2	205
**50**	107.9 ± 0.6	247	106.7 ± 0.7	162	101.2 ± 0.6	224	110 ± 1	194
**100**	121.6 ± 0.4	170	114 ± 0.7	140	107.7 ± 0.9	134	107.1 ± 0.9	275
**200**	130 ± 1	241	121 ± 2	119	120 ± 0.9	222	94 ± 1	225
**400**	147.5 ± 0.6	165	135 ± 1	121	142 ± 1	119	88.5 ± 0.4	259
**800**	147.6 ± 0.5	221	140.3 ± 0.8	168	150.4 ± 0.9	145	86.8 ± 0.3	284
**1600**	165.6 ± 0.6	111	144.6 ± 0.7	247	163.7 ± 0.6	123	112 ± 2	212

SI Table 4. The RMS values of HU-DNA in presence of PEG8000 in buffer containing and absence of $MgCl_2$.

HU Conc. (nM)	3 % PEG8000 without $MgCl_2$	N	9 % PEG8000 without $MgCl_2$	N	15 % PEG8000 without $MgCl_2$	N	3 % PEG8000 without $MgCl_2$	N
**0**	144.8 ± 0.6	214	155 ± 1	194	154 ± 1	187	144 ± 2	192
**6.25**	146.1 ± 0.6	271	152.3 ± 0.5	231	151.4 ± 0.5	286	150.3 ± 0.9	292
**12.5**	146.1± 0.9	164	154.8± 0.6	200	144.1 ± 0.7	363	145.6 ± 0.9	
**25**	142.3 ± 0.7	227	143 ± 1	156	118 ± 1	147	145.1 ± 0.7	278
					88 ± 1	146		
**50**	142 ± 1	123	109.6 ± 0.6	273	107 ± 2	173	105 ± 1	125
					71 ± 2	275		
**100**	117.7 ± 0.9	199	102.2 ± 0.4	260	104 ± 3	210	90.2 ± 2	229
					43.1 ± 0.4	246		
**200**	104.3 ± 0.7	343	106.3± 0.4	299	97 ± 0.8	147	97 ± 2	143
					41 ± 1	251		
**400**	143 ± 1	445	133 ± 2	176	122 ± 0.7	---	149 ± 1	111
**800**	159 ± 1	144	141.2 ± 0.8	225	---	---	157.5 ± 0.4	91
**1600**	---	---	---	---	---	---	117.4 ± 0.5	438

SI Table 5. Fitting values of Hill function.

Conditions	Compaction regime	Extension regime
**Without $MgCl_2$**	---	2.5 ± 0.6
**With 8 mM $MgCl_2$**	---	4.9 ± 0.4
**0.5 % BGB**	3.3 ± 0.9	2.4 ± 0.3
**1 % BGB**	2.6 ± 0.7	2.5 ± 0.3
**1.25 % BSA**	1.7 ± 0.5	1.4 ± 0.2
**5 % BSA**	2.0 ± 0.7	2.0 ± 0.2
**10 % BSA**	1.6 ± 0.6	1.6 ± 0.2
**3 % PEG8000**	5.0 ± 0.8	6 ± 5
**9 % PEG8000**	4.3 ± 0.2	4.9 ± 0.1
**15 % PEG8000**	10 ± 5	35± 0
**0.5 % BGB with $MgCl_2$**	1.8 ± 0.5	2.7 ± 0.7
**10 % BSA with $MgCl_2$**	1.2 ± 0.2	---
**3 % PEG8000 with $MgCl_2$**	1.8 ± 0.7	2.7 ± 0.7
